# Optimizing Human Synovial Fluid Preparation for Two-Dimensional Gel Electrophoresis

**DOI:** 10.1186/1477-5956-9-65

**Published:** 2011-10-11

**Authors:** Carl PC Chen, Chih-Chin Hsu, Wen-Lin Yeh, Hsiu-Chu Lin, Sen-Yung Hsieh, Shih-Cherng Lin, Tai-Tzung Chen, Max JL Chen, Simon FT Tang

**Affiliations:** 1 Department of Physical Medicine & Rehabilitation Chang Gung Memorial Hospital, Linkou and Chang Gung University College of Medicine, Tao-Yuan County, Taiwan; 2 Department of Physical Medicine & Rehabilitation Chang Gung Memorial Hospital, Keelung and Chang Gung University College of Medicine, Tao-Yuan County, Taiwan; 3 Department of Orthopedic Surgery Chang Gung Memorial Hospital, Linkou and Chang Gung University College of Medicine, Tao-Yuan County, Taiwan; 4 Clinical Proteomics Core Laboratory, Chang Gung Memorial Hospital, Linkou, Taiwan

## Abstract

**Background:**

Proteome analysis is frequently applied in identifying the proteins or biomarkers in knee synovial fluids (SF) that are associated with osteoarthritis and other arthritic disorders. The 2-dimensional gel electrophoresis (2-DE) is the technique of choice in these studies. Disease biomarkers usually appear in low concentrations and may be masked by high abundant proteins. Therefore, the main aim of this study was to find the most suitable sample preparation method that can optimize the expression of proteins on 2-DE gels that can be used to develop a reference proteome picture for non-osteoarthritic knee synovial fluid samples. Proteome pictures obtained from osteoarthritic knee synovial fluids can then be compared with the reference proteome pictures obtained in this study to assist us in identifying the disease biomarkers more correctly.

**Results:**

The proteomic tool of 2-DE with immobilized pH gradients was applied in this study. A total of 12 2-DE gel images were constructed from SF samples that were free of osteoarthritis. In these samples, 3 were not treated with any sample preparation methods, 3 were treated with acetone, 3 were treated with 2-DE Clean-Up Kit, and 3 were treated with the combination of acetone and 2-D Clean-Up Kit prior to 2-DE analysis. Gel images were analyzed using the PDQuest Basic 8.0.1 Analytical software. Protein spots that were of interest were excised from the gels and sent for identification by mass spectrometry. Total SF total protein concentration was calculated to be 21.98 ± 0.86 mg/mL. The untreated SF samples were detected to have 456 ± 33 protein spots on 2-DE gel images. Acetone treated SF samples were detected to have 320 ± 28 protein spots, 2-D Clean-Up Kit treated SF samples were detected to have 413 ± 31 protein spots, and the combined treatment method of acetone and 2-D Clean-Up Kit was detected to have 278 ± 26 protein spots 2-DE gel images. SF samples treated with 2-D Clean-Up Kit revealed clearer presentation of the isoforms and increased intensities of the less abundant proteins of haptoglobin, apolipoprotein A-IV, prostaglandin-D synthase, alpha-1B-glycoprotein, and alpha-2-HS-glycoprotein on 2-DE gel images as compared with untreated SF samples and SF samples treated with acetone.

**Conclusions:**

The acetone precipitation method and the combined treatment effect of acetone and 2-DE Clean-Up Kit are not preferred in preparing SF samples for 2-DE analysis as both protein intensities and numbers decrease significantly. On the other hand, 2-D Clean-Up Kit treated SF samples revealed clearer isoforms and higher intensities for the less abundant proteins of haptoglobin, apolipoprotein A-IV, prostaglandin-D synthase, alpha-1B-glycoprotein, and alpha-2-HS-glycoprotein on 2-DE gels. As a result, it is recommended that SF samples should be treated with protein clean up products such as 2-D Clean-Up Kit first before conducting proteomic research in searching for the relevant biomarkers associated with knee osteoarthritis.

## Background

In recent years, proteome analysis is frequently applied in clinical diagnostics and predictive medicine[1]. Although it can be technically challenging, it is the preferred analytical method in which a large number of proteins (biomarkers) can be identified. Two-dimensional gel electrophoresis (2-DE) with immobilized pH gradients (IPGs) is an effective tool in proteomics in which hundreds of proteins can be identified simultaneously[2]. Presently, there are numerous studies which apply 2-DE analysis on body fluids such as cerebrospinal fluid (CSF), synovial fluid (SF), and plasma to explore the biomarkers that are related to aging and knee osteoarthritis (OA)[3].

OA is a common chronic degenerative joint disease. Although clinical research on OA has been extensively explored, the etiology of this disease is still unclear [4]. Proteomics have been applied to discover the biomarkers that may be beneficial in the diagnosis, prognosis, and treatment of OA [5]. Disease biomarkers usually appear in low concentrations and may be masked by high abundant proteins [6]. A practical thing to do will be to remove the unwanted and uninformative high abundant proteins, thus enhancing the disease related low abundant proteins. However, some studies have mentioned that the removal of abundant proteins may also remove some of the less abundant proteins[7].

In this study, sample preparation methods of acetone precipitation and 2-DE Clean-Up Kit will be applied to human knee joint SF to examine which method is most effective in enhancing protein spots on 2-DE gel images and in removing the abundant proteins while preserving the low-abundant proteins that may be of interest to us. Acetone precipitation method was discovered to be the most effective way in optimizing body fluids with low protein concentration and high salt content such as CSF to enhance their protein presentation on 2-DE gel images [8]. SF is secreted by the synovial cell lining and is a transudate of plasma. It has higher protein concentration as compared with CSF, and does not have high salt content [9]. As a result, the acetone precipitation method may not be needed to treat the SF samples in order to enhance the SF protein presentation on 2-DE gel images. The influence of the acetone precipitation method on the presentation of SF proteins on 2-DE gel images has never been investigated in other literature. The combined effect of acetone and 2-DE Clean-Up Kit on the presentation of SF proteins on 2-DE gel images also has never been investigated.

In this study, the acetone precipitation method, 2-DE Clean-Up Kit, and their combined effect when used together to treat the SF samples will be explored thoroughly to investigate their influence on the presentation of SF proteins on 2-DE gel images. We hypothesize that the 2-DE Clean-Up Kit should be more effective in contributing to the presentation of SF protein spots on 2-DE gel images and in the removal of abundant proteins from SF samples as compared with the acetone precipitation method for the analysis and detection of the less abundant proteins.

## Materials and methods

### Subjects and Sample Gathering

A total of 6 patients (4 males and 2 females, with an average age of 48 years) diagnosed with visible fluid accumulation at the knee supra-patellar bursa areas were recruited in this study. All patients signed the informed consent before participating in this study. The institutional ethics committee approved all the protocols involved in this study. They were free of knee joint disorders such as OA. Musculoskeletal ultrasound was used to precisely identify the location of fluid accumulation site, and subsequent ultrasound-guided aspiration of the SF was performed. Approximately 5 to 10 mL of SF was aspirate from each patient and then aliquoted subsequently. The aliquoted samples were stored at -20°C if they were to be used for experimental analysis within one week. Otherwise the samples were stored at -80°C. SF samples from all 6 patients were used for the calculation of SF total protein concentrations.

### The Calculation of Synovial Fluid Total Protein Concentrations

The standard curve of known bovine serum albumin (BSA) (Sigma, > 96% purity) concentrations (2 μg, 1 μg, 0.5 μg, 0.25 μg, and 0.125 μg) was constructed to calculate the total protein concentrations of the SF. The Bradford method was applied for the calculation of protein concentrations[10]. The Bio-Rad Protein Assay dye reagent was used. The dye-proteins were measured by Unicam UV1 spectrophotometer at an absorbance of 595 nm. The SF samples were diluted 30 times first by ddH_2_O in order to fit into the linear BSA standard curve for protein concentration calculation.

### Sample Preparation Methods for 2-DE

All the SF samples were vortexed first and then centrifuged at a slow speed of 1,000 rpm before the samples were aspirated for further experimental purposes. One mL of SF was aspirated from the SF samples of all the 6 patients, and then mixed together. The mixture was then vortexed and centrifuged at a slow speed of 1,000 rpm. Two-DE gels were constructed from the SFs aspirated from this mixed pool of SF samples from all 6 patients.

Fifteen μL, equivalent to approximately 300 μg of total protein concentration, was aspirated from the pooled SF sample for the construction of 2-DE gels. Triplicates of 2-DE gels were constructed for the untreated SF samples, SF samples treated with acetone precipitation method [8], SF samples treated with acetone first and then subsequently by the 2-D Clean-Up-Kit, and SF samples treated with 2-D Clean-Up-Kit (GE Healthcare Bio-Science Corp., Piscataway, USA) for a total of 12 2-DE gels. The SF treatment protocols were:

A. Fifteen μL of pure untreated SF sample was mixed thoroughly with 450 μL of rehydration buffer (8 M Urea, 0.5% CHAPS, 0.2% DTT, 0.2% Pharmalyte, pH 3-10) and before loading onto the 17-cm IPG strip (BioRad).

B. Acetone precipitation method: 15 μL of SF was mixed with 1000 μL of acetone and incubated on ice for a total of 60 minutes. The mixture was then centrifuged at 12,000 × g for 30 minutes. After centrifuging, the supernatant was discarded, and the precipitate was left air-dried for about 10 minutes. The precipitate was then re-dissolved using 450 μL of rehydration buffer before loading onto the 17-cm IPG strip.

C. 2-D Clean-Up Kit: Briefly, 15 μL of SF sample was mixed with three volumes of manufacturer's precipitant and co-precipitant, and then incubated on ice for 15 minutes. The mixture was then centrifuged at 8000 × g for 10 minutes. After discarding the supernatant, the precipitate was washed by 1 mL of wash buffer and 5 μL wash additive at - 20°C for 30 minutes. After the mixture was further centrifuged at 12,000 × g for 5 minutes, the supernatant was discarded and the precipitate was left air-dry for about 10 minutes. The precipitate was then re-dissolved using 450 μL of rehydration buffer before loading onto the 17-cm IPG strip.

D. Combined sample treatment method of acetone and 2-D Clean-Up-Kit: The 15 μL of SF sample was treated with acetone precipitant method first. After obtaining the SF precipitate from the acetone precipitant method, three volumes of manufacturer's precipitant and co-precipitant solutions from 2-D Clean-Up-Kit were then added to the precipitate. The remaining treatment steps were similar to the 2-D Clean-Up-Kit protocol mentioned above.

### 2-DE

Iso-electric focusing (IEF) was performed using 17-cm, p*I *3-10 non-linear IPG strips. The rehydration buffer containing the untreated SF or protein precipitate (after treatment with acetone, 2-D Clean-Up-Kit or the combination of both) were loaded onto the IPG strips and rehydrated overnight (in-gel rehydration method) at room temperature in a reswelling tray. The strips were focused at 50 μA/IPG strip for a total of 60 kVh at 20°C using the Protean IEF Cell (BioRad). After focusing, two equilibration steps were performed (1% DTT for the first step and 4.8% iodoacetamide for the second step), each step for 15 min. Following equilibration, IPG strips were washed with SDS-polyacrylamide (SDS-PAGE) running buffer and then applied onto the top of 12% SDS-PAGE gels. The strips were then sealed with 0.5% molten agarose solution. The second dimension separation in the second dimension was carried out using the Protean II electrophoresis equipment and Tris-glycine buffer (25 mM Tris, 192 mM glycine) containing 0.1% SDS. The running condition was set at constant 40 mA per gel and with the temperature at 10°C. Running was completed until the bromophenol blue dye front reached the bottom of each gel. Gels were then placed in a fixing solution (containing 50% methanol and 7% acetic acid) overnight in preparation for protein visualization using SYPRO Ruby Protein Gel Stain (Molecular Probes, Invitrogen). Staining procedures were followed according to the Basic Protocol product information manual. In this study, 12 good quality SYPRO Ruby stained SF 2-DE gel images were obtained (3 gel images of pure non-treated SF samples, 3 gel images of SF treated with acetone, 3 gel images of SF treated with 2-D Clean-Up Kit, and 3 gel images of SF samples treated with acetone first and then subsequently by the 2-D Clean-Up-Kit).

### Image Analysis

The ProXPRESS™ 2D Proteomic Image System (PerkinElmer Life and Analytical Sciences) was used to scan the SYPRO Ruby stained SF 2-DE gels. The scanner was set with an excitation of 520 nm, and emission filter of 610BP30 as recommended by the product protocol. The gel images were acquired as digital TIF files and analyzed using the PDQuest Basic 8.0.1 Analytical software (BioRad).

### Protein Identification by Mass Spectrometry (MS)

Protein spots of interest were manually excised with a tip (approximately 1~2 mm in diameter) from the SYPRO Ruby stained SF 2-DE gels and was destained using 50 mM NH4HCO3 in 50% ACN and dried in a SpeedVac concentrator. The protein was then digested by incubating overnight at 37°C with trypsin (Promega, Madison, WI; at 5 ng/mL) in 50 mM NH4HCO3, pH 7.8. Tryptic peptides were extracted from the gel pieces in 1 volume of 0.1% TFA, while vortexing for 5 minutes, followed by sonication for 5 minutes. Crude digest mixtures were concentrated and desalted using mC18 ZipTips (Millipore) followed by eluting in 1.5 mL of matrix (5 mg of CHCA/mL in 50% ACN/0.1% TFA) for MALDI-TOF MS and MS/MS analyses. Both MS and MS/MS spectra were searched against the NCBI database, using MASCOT software from matrix science (http://www.matrixscience.com), to identify the proteins. The MALDI-TOF MS resolution for the peptides was around 20,000, and the mass accuracy was 0.01-0.02 Da.

### Data Analysis

The numbers of protein spots were detected by the PDQuest Basic 8.0.1 Analytical software from the constructed 12 2-DE gel images. The SF protein concentrations, and the numbers of protein spots from untreated SF sample, and samples treated with acetone and/or 2-D Clean-Up Kit were expressed as mean ± standard error of means (SEM). ANOVA and *t*-test were used as appropriate to compare the means from different SF sample treatments. The Statistical Program for Social Sciences (SPSS) version 13 (SPSS Inc., Chicago) and Excel 2007 (Windows Office 2007) were used for data calculations. Values of *p *< 0.05 were considered statistically significant.

## Results

The SF samples had to be diluted by 30 times in order for the measured optical density (OD) values to fit into the linear standard curve of OD value versus known BSA concentrations for total protein concentration calculations. The SF total protein concentration was calculated to be 21.98 ± 0.86 mg/mL.

After image analyses, the untreated SF sample was detected to have an average of 456 ± 33 protein spots on 2-DE gels. SF sample treated with acetone precipitation method was detected to have an average of 320 ± 28 protein spots, and SF sample treated with 2-D Clean-Up Kit was detected to have an average of 413 ± 31 protein spots on 2-DE gels. SF sample treated with acetone first and then subsequently with 2-D Clean-Up Kit was detected to have an average of 258 ± 26 protein spots on 2-DE gels. In summary, SF samples treated with acetone precipitation method and with acetone first followed by 2-D Clean-Up Kit revealed significantly less protein spots on 2-DE gels as compared with untreated SF, and SF treated with 2-D Clean-Up Kit. After treating SF sample with acetone, further treatment with 2-D Clean-Up Kit resulted in further reduction in the number of protein spots and protein concentration (Table 1).

**Table 1:** Number of detected protein spots on the 2-DE gel images.

SF Sample Treatment Method (n = 3)	Number of Protein Spots
Untreated	456 ± 33
Acetone Precipitation	320 ± 28^+#^
2-D Clean-Up Kit	413 ± 31
Acetone and 2-D Clean-Up Kit	278 ± 26*

Comparison between acetone precipitation method and untreated SF sample: + = *p *< 0.05.

Comparison between acetone precipitation method and 2-D Clean-Up Kit: # = *p *< 0.05.

Comparison between combined acetone/2-D Clean-Up Kit treated SF sample, and untreated

SF sample, and SF sample treated with acetone precipitation method: * = *p *< 0.05.

Values expressed as mean ± standard error of means (SEM).

Protein assay was performed again to measure the total protein concentrations in the precipitates obtained after SF sample treatments. The SF total protein concentration after treatment with acetone precipitation method was about 15.79 ± 0.79 mg/mL, with a significant protein loss of about 22 ± 8% as compared with the untreated SF. After SF sample treatment with 2-D Clean-Up Kit, the total protein contraction was 19.33 ± 0.98 mg/mL, with an insignificant protein loss of about 14 ± 7% as compared with the untreated SF. SF sample treatment using acetone first and then subsequently by 2-D Clean-Up kit decreased the total protein concentration further to 13.41 ± 0.55 mg/mL, which was significantly less as compared with untreated SF, and SF sample treated with 2-D Clean-Up Kit (*p *< 0.01). The observed protein loss when treating the acetone treated SF sample further with 2-D Clean-Up Kit was significant (15.79 ± 0.79 mg/mL => 13.41 ± 0.55 mg/mL, *p *< 0.05) (Table 2).

**Table 2:** Comparisons of total protein concentrations and their percentages of protein loss after different SF sample treatment methods

SF Sample Treatment	Total Protein Concentration in mg/mL (n = number of samples used)	Percentage of Total Protein Loss (%)
Untreated	21.98 ± 1.66 (n = 6)	
Acetone Precipitation	15.79 ± 0.79 (n = 3)^+#^	22 ± 8
2-D Clean-Up Kit	19.33 ± 0.98 (n = 3)	14 ± 7
Acetone and 2-D Clean-Up Kit	13.41 ± 0.55 (n = 3)^‡^*	39 ± 5

Comparison between acetone precipitation method and untreated SF sample: + = *p *< 0.05.

Comparison between acetone precipitation method and 2-D Clean-Up Kit: # = *p *< 0.05.

Comparison between combined acetone/2-D Clean-Up Kit treated SF sample, and 2-D Clean-Up Kit treated SF sample: ‡ = *p *< 0.05.

Comparison between combined acetone/2-D Clean-Up Kit treated SF sample, and untreated SF sample, and SF sample treated with acetone precipitation method: * = *p *< 0.01.

Values expressed as mean ± standard error of means (SEM).

**Figure 1:**
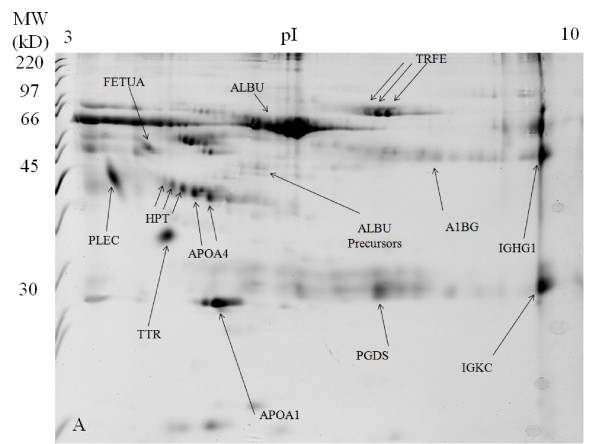
**SYPRO Ruby stained 2-DE gel image of the untreated knee synovial fluid sample**.

**Figure 2:**
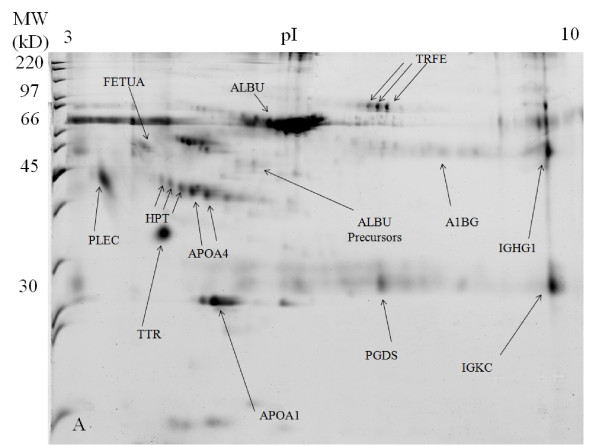
**SYPRO Ruby stained 2-DE gel image of the knee synovial fluid sample treated with acetone precipitation method**.

**Figure 3:**
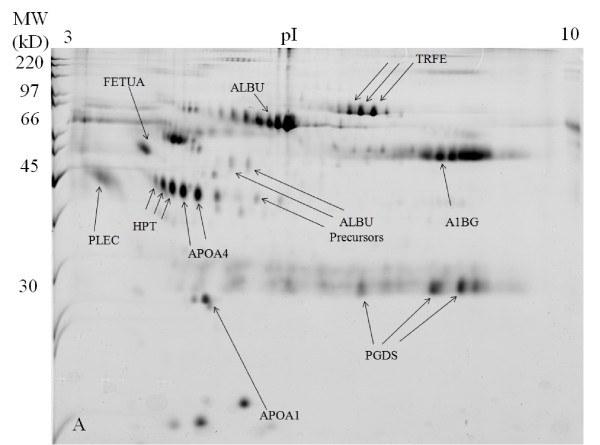
**SYPRO Ruby stained 2-DE gel image of the knee synovial fluid sample treated with 2-D Clean-Up Kit**.

**Figure 4:**
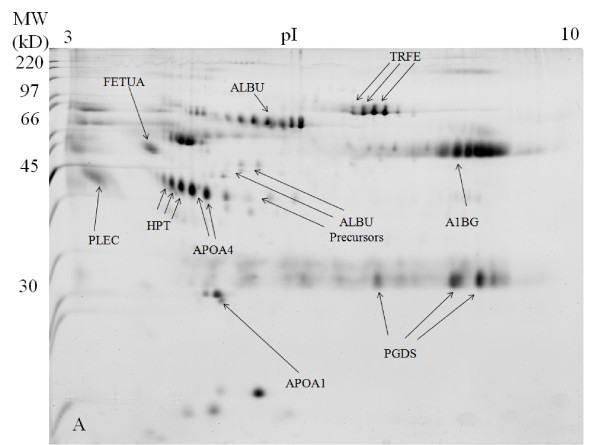
**SYPRO Ruby stained 2-DE gel image of the knee synovial fluid sample treated with acetone precipitation method first and then subsequently with 2-D Clean-Up Kit**.

In the untreated SF 2-DE gel images, the high abundant protein of albumin appeared as a huge lump of protein from p*I *values of approximately 4 to 7 at 66 kDa, with its isoforms appearing blurry and not well separated from each other (Figure 1). The 2-DE gel images of the acetone treated SF sample appeared quite similar to the 2-DE gels images of the untreated SF sample except that the overall intensity is lower (Figure 2). After SF sample treatment with 2-D Clean-Up Kit, the huge albumin lump and the other abundant protein of immunoglobulin gamma-1 chain C region protein have disappeared. Albumin isoforms became more clearly visible and well separated from each other. Although there was total protein concentration loss of about 15% after sample treatment with 2-D Clean-Up Kit, there was no evidence of depletion of less abundant proteins. The intensities of non-abundant proteins of haptoglobin (HPT), apolipoprotein A-IV (APOA4), prostaglandin-D synthase (PGDS), alpha-1B-glycoprotein (A1BG), and alpha-2-HS-glycoprotein (FETUA) have increased, indicating that the concentrations of these proteins have actually increased as compared with the untreated SF gel images. The isoforms of these proteins have become more clearly visible and well separated from each other. Taking APOA4 as an example, its total protein intensity increased by 20% in the 2-D Clean-Up Kit treated SF sample as compared with the untreated SF sample (Figure 3). The 2-DE gel images of SF sample treated with acetone first and then subsequently with 2-D Clean-Up Kit appeared similar to the 2-DE gels images of the SF sample treated with 2-D Clean-Up Kit except that the overall intensity is lower, indicating a further decrease of about 17% in protein concentration (Figure 4).

Not all non-abundant proteins revealed increases in overall intensities and in the number of isoforms after SF sample treatment with 2-D Clean-Up Kit. Transthyretin (TTR) disappeared from the 2-DE gel images after sample treatment with 2-D Clean-Up Kit. The intensities of the protein spots of plectin and apolipoprotein A-1 (APOA1) decreased by about 20% as compared with the untreated SF samples. The names of the protein spots identified in this study confirmed by MS and subsequently by the SWISS-PROT database are listed in Table 3.

**Table 3:** Twelve representative proteins from human SF identified by MS and separated by 2 - DE

Spot name^a^	Description	Access No^a^	Mr(kDa)^b^	pI^b^	No. of Matched^c^	Seq Cov (%)^d^
A1BG	Alpha-1B-glycoprotein	P04217	54.79	5.56	15	30
ALBU	Serum albumin	P02768	71.32	5.92	27	54
APOA1	Apolipoprotein A-1	P02647	30.00	5.56	13	46
APOA4	Apolipoprotein A-IV	P06727	45.30	5.28	22	50
FETUA	Alpha-2-HS-glycoprotein	P02765	40.00	5.43	10	38
HPT	Haptoglobin	P00738	45.80	6.13	20	44
IGHG1	Ig gamma-1 chain C region	P01857	36.60	8.46	8	30
IGKC	Ig kappa chain C region	P01834	11.77	5.58	4	64
PGDS	Prostaglandin-H2 D-isomerase	P41222	21.01	7.66	12	55
TRFE	Serotransferrin	P02787	79.29	6.81	24	36
TTHY	Transthyretin	P02766	15.87	5.52	10	73

a. Protein name and accession number according to the SWISS-PROT databases.

b. Predicted molecular weights in kilodaltons and p*I *on SWISS-PROT database.

c. Number of peptide masses matching the top hit from MS-Fit PMF.

d. The percentage of sequence coverage of matched peptides in the identified protein.

## Discussion

Compared to other body fluid, SF can be obtained quite readily from the knee joints. With real-time imaging support from musculoskeletal ultrasound, SF can be precisely aspirated from knee joints to avoid blood contamination [11]. The proteome analysis of the SF can help us understand the molecular mechanisms behind the onset, progression, and treatment effectiveness of knee OA [5]. As a result, finding the most suitable sample treatment method that can enhance the presentation of the less abundant protein spots and their isoforms on 2-DE gels can certainly be beneficial to OA research [6]. In this study, the influences of acetone and 2-D Clean-Up Kit, and the combination of both sample treatment methods on SF 2-DE gel protein presentations were compared. The SYPRO Ruby staining method was chosen as it has good linearity for data comparison as compared with other staining methods such as coomasie blue and silver stain [12].

Albumin and immunoglobulin G constitute about 65% and 15% of total protein concentrations in the plasma respectively[6]. The identification of potential less abundant disease biomarkers may be complicated or masked by these highly abundant proteins [7]. As a result, finding an efficient method that can remove these abundant proteins and not jeopardizing the presentation of less abundant proteins on 2-DE gel images is important in disease biomarker research. There are numerous kits and spin columns that can be purchased to remove abundant proteins from samples. The 2-D Clean-Up Kit was chosen in this study as it was proven to have better protein recoveries and clearer backgrounds on 2-DE gel images as compared with other products [8]. The 2-D Clean-Up Kit was designed to prepare samples that are too dirty or too dilute for 2-DE. The reagents quantitatively precipitate proteins while leaving interfering substances, such as detergents, salts, lipids, phenolics, and nucleic acids in the supernatant solution to be discarded (GE Healthcare product user manual).

In this study, although there was a decrease of about 15% in total protein concentration in 2-D Clean-Up Kit treated SF samples, image analytical software did not reveal significant differences in the number of protein spots as compared with untreated SF samples. Some literature has mentioned that the removal of abundant proteins may decrease the number or influence the pattern of less abundant proteins on 2-DE gels images[7]. This was not observed in this study. In fact, after treating SF samples with 2-D Clean-Up Kit, protein spots such as HPT, APOA4, PGDS, A1BG, and FETUA showed increases in intensities as compared with the untreated SF samples. The isoforms of HPT and APOA4 protein spots became clearly visible and well separated from each other with the overall protein intensity of APOA4 increased significantly by about 20%. More protein spots were visible in the 30 kDa range, such as the less abundant protein of PGDS and its isoforms. In the high abundant protein of albumin, clear and well separated albumin isoforms, instead of a large albumin lump, were visible from p*I *values of approximately 4 to 7 after sample treatment with 2-D Clean-Up Kit. A higher number of albumin precursors were also visible.

A study by Chen and colleagues has shown that treating CSF samples with 2-D Clean-Up Kit wipes out the protein spot of TTR from 2-DE gel images [8]. This phenomenon was observed in our SF samples as well. TTR disappeared from 2-DE gel images after SF sample treatment with 2-D Clean-Up Kit. The protein spots of plectin and APOA1 showed decreases in protein intensities of about 20% in the 2-D Clean-Up Kit treated SF samples as compared with the untreated SF samples. As a result, we must be careful in interpreting the protein concentrations of the non-abundant protein spots such as TTR, plectin, and APOA1 on 2-DE gels in OA research as they may disappear or decrease in intensity levels after sample treatment with protein removal products.

The acetone precipitation method was shown to have high protein recovery percentage and enhancing the presentation of protein spots on 2-DE gel images when used in treating body fluids with low protein concentrations such as CSF[8]. This may be true for CSF as it has high salt content, and extremely low protein concentration (about 0.4 mg/mL as compared with plasma protein concentration of 55 mg/mL)[13,14]. Acetone can be used to wash away the salt content, and concentrating the CSF proteins that are too dilute for effective 2-DE analysis [8,15]. However, acetone cannot wash away the highly abundant proteins as shown by the presence of albumin lump and heavy chain immunoglobulin G on 2-DE gel images. SF has a much higher protein concentration (measured to be 21.98 ± 0.86 mg/mL in this study) as compared with CSF. As a result, SF has adequate protein concentration for effective 2-DE analysis without needing acetone to concentrate its proteins. In fact, the acetone precipitation method is not preferred for SF samples as it washes away approximately 22% of the total protein concentration, causing the overall protein intensities on 2-DE gel images to decrease. The presentation of the less abundant protein spots and their isoforms also became blurry in acetone treated SF samples as compared with 2-D Clean-Up Kit treated and untreated SF samples. In this study, SF sample was further treated with 2-D Clean-Up Kit after treating with acetone precipitation method first. This combination of SF sample treatment methods has not been reported in other literature before. The 2-DE gel images were similar to the images of the SF samples treated with 2-D Clean-Up Kit but with lower intensities of the less abundant protein spots. There was also a loss in the total protein concentration of about 40% as compared with the untreated SF samples. As a result, we do not recommend this combination of SF treatment methods in treating SF samples for 2-DE analysis.

In OA research, large quantitative variability among protein spot presentations on 2-DE gel images in SF samples of knee OA patients can be observed [6]. As a result, it is crucial to develop a reference proteome picture for the non-OA knee SF proteins. In this study, 2-DE gel images were constructed from SF samples obtained from patients without knee OA. Good reproducibility was observed in all the 2-DE gel images (>90% gel matching rate, data not presented). Other OA related studies can compare their gel images with the reference proteome pictures obtained in this study to assist them in differentiating whether the observed protein patterns on 2-DE gel images are due to OA or not. For instance, an increase in the intensity of APOA4 protein by 10% on 2-DE gel images of OA knee SF after treating with 2-DE Clean-Up Kit or other related products does not mean that APOA4 can be used a potential biomarker in OA. As observed in this study, an increase of APOA4 intensity by 20% was evident in non-OA SF samples after sample treatment with 2-D Clean-Up Kit. The 2-DE gel image in Figure 1 obtained in this study may be used as a reference image for SF samples not treated with any preparation methods. Gel image obtained in Figure 3 may be used as a reference image for OA research on the study of less abundant proteins in knee SF samples.

## Conclusions

In this study, proteomic approach was performed in finding the suitable SF sample preparation method for effective 2-DE analysis. The acetone precipitation method is not suitable for SF samples as it will cause a decrease in total protein concentration by about 22% and a decrease in overall protein intensity on 2-DE gel images. SF samples treated with 2-D Clean-Up Kit showed increases in the intensities of the less abundant proteins of HPT, APOA4, PGDS, A1BG, and FETUA and with their isoforms more clearly separated on 2-DE gel images as compared with the images from untreated SF and SF samples treated with acetone. The combined treatment method of acetone and 2-D Clean-Up Kit is also not recommended for treating SF samples as it further decreases the total protein concentrations, intensities of protein spots, and the number of protein spots on 2-DE gels. As a result, it is recommended that knee SF samples should be treated with protein removal products such as the 2-D Clean-Up Kit first before conducting proteomic 2-DE biomarker research in OA knee SF samples.

## Abbreviations

2-DE: 2-dimensional electrophoresis; BSA: bovine serum albumin; IPGs: immobilized pH gradients; IEF: iso-electric focusing; kDa: kilodaltons; μg: microgram; μL: microliter; mg: milligram; mL: milliliter; nm: nanometer; SDS-PAGE: sodium dodecyl sulfate polyacrylamide gel electrophoresis; TTR: transthyretin.

## Competing interests

The authors declare that they have no competing interests.

## Authors' contributions

CPC performed the experiments, participated in the design of the study and the statistical analysis, and drafted the manuscript. CCH participated in statistical analysis. WLY helped in providing the synovial fluid samples. HCL performed the in-gel digestion and mass spectrometry protein identification. SYH is the director of the proteomics core lab and provided technical guidance to this study. SCL helped with the drawing of the figures. TTC performed the protein assay experiments. MJL assisted in the literature review of this study. SFT participated in the design of this study.

All authors read and approved the final manuscript.
